# Intermuscular coherence reveals that affective emotional pictures modulate neural control mechanisms during the initiation of arm pointing movements

**DOI:** 10.3389/fnhum.2023.1273435

**Published:** 2024-01-05

**Authors:** Emeline Pierrieau, Camille Charissou, Sylvie Vernazza-Martin, Benjamin Pageaux, Romuald Lepers, David Amarantini, Lilian Fautrelle

**Affiliations:** ^1^ToNIC, Toulouse NeuroImaging Center, Université de Toulouse, Inserm, Paul Sabatier University, Toulouse, France; ^2^Aquitaine Institute for Cognitive and Integrative Neuroscience (INCIA), Université de Bordeaux, Bordeaux, France; ^3^Institut National Universitaire Champollion, EIAP, Département STAPS, Rodez, France; ^4^Université Paris Nanterre, UFR-STAPS, Nanterre, France; ^5^Laboratoire des interactions Cognition, Action, Émotion - LICAÉ, UFR STAPS, Université Paris Nanterre, Nanterre, France; ^6^Centre de recherche de l'Institut universitaire de gériatrie de Montréal (CRIUGM), Montréal, QC, Canada; ^7^École de kinésiologie et des sciences de l'activité physique (EKSAP), Faculté de médecine, Université de Montréal, Montréal, QC, Canada; ^8^Centre interdisciplinaire de recherche sur le cerveau et l'apprentissage (CIRCA), Montréal, QC, Canada; ^9^CAPS UMR1093, Institut National de la Santé et de la Recherche Médicale (INSERM), Faculté des Sciences du Sport, Université de Bourgogne-Franche-Comté, Dijon, France

**Keywords:** intermuscular coupling, IAPS, motor control, EMG, emotional context

## Abstract

**Introduction:**

Several studies in psychology provided compelling evidence that emotions significantly impact motor control. Yet, these evidences mostly rely on behavioral investigations, whereas the underlying neurophysiological processes remain poorly understood.

**Methods:**

Using a classical paradigm in motor control, we tested the impact of affective pictures associated with positive, negative or neutral valence on the kinematics and patterns of muscle activations of arm pointing movements performed from a standing position. The hand reaction and movement times were measured and electromyography (EMG) was used to measure the activities from 10 arm, leg and trunk muscles that are involved in the postural maintenance and arm displacement in pointing movements. Intermuscular coherence (IMC) between pairs of muscles was computed to measure changes in patterns of muscle activations related to the emotional stimuli.

**Results:**

The hand movement time increased when an emotional picture perceived as unpleasant was presented as compared to when the emotional picture was perceived as pleasant. When an unpleasant emotional picture was presented, beta (β, 15–35 Hz) and gamma (γ, 35–60 Hz) IMC decreased in the recorded pairs of postural muscles during the initiation of pointing movements. Moreover, a linear relationship between the magnitude of the intermuscular coherence in the pairs of posturo-focal muscles and the hand movement time was found in the unpleasant scenarios.

**Discussion:**

These findings reveal that emotional stimuli can significantly affect the content of the motor command sent by the central nervous system to muscles when performing voluntary goal-directed movements.

## 1 Introduction

From a psychological framework, two main theoretical models are usually proposed to explain the way in which emotional contexts may modulate motor performance. The first is the motivational direction model (Lang, 1995; Cacioppo and Gardner, 1999; Fishbach and Shah, 2006), while the second is the affective meaning of the intended response goal model (Haggard, 2005; Shadmehr, 2010; Haith et al., 2012). According to the first hypothesis, the emotional context in which voluntary movements are performed would stimulate motivations for approach or avoidance (Lang et al., 1990; Lang, 1995). According to the second model, the motor behavioral consequences, rather than the emotional valences of the stimuli themselves, significantly modulate the contents of the voluntary motor command generated by the central nervous system (CNS) (Shadmehr, 2010; Vernazza-Martin et al., 2017). Both models show a link between emotional contexts and motor performance, providing evidence that viewing pictures perceived as pleasant or unpleasant during the same voluntary motor task can modulate the movement produced (Solarz, 1960; Naugle et al., 2010; Phaf et al., 2014; Yiou et al., 2014; Vernazza-Martin et al., 2017).

Nevertheless, the available empirical evidence does not align consistently with this hypothesis as the effects of valenced stimuli on behavioral reactions are extremely mixed and do not reveal a reproducible pattern (Mancini et al., 2020, 2022; Mirabella et al., 2022). The considerable heterogeneity of results can be accounted for by the fact that the impact of emotional information on behavior is not fixed. Rather, it is closely linked to the relevance of the stimuli in a specific context, as suggested by the appraisal theories of emotion (Moors and Fischer, 2019; Scherer and Moors, 2019). Some recent studies showed emotional facial expressions (Mirabella, 2018; Mancini et al., 2020, 2022; Mirabella et al., 2020, 2022) and body postures (Calbi et al., 2022) elicit a consistent, replicable behavioral effect only when they are relevant to participants' goals, i.e., the effect of emotions is context-dependent. Dealing with forward gait initiation, Mirabella et al. (2022) showed that whole-body movements share the same features as reaching arm movements regarding emotional stimuli, i.e., facial emotions were able to modulate movement parameters when their conscious appraisal was requested.

Altogether, these findings corroborate the idea that, in some cases, internal emotional state could modulate the neural mechanisms of the muscular command for a given action (Frijda, 1986). Consequently, the present study proposes to address this idea through a novel and multidisciplinary approach combining validated tools from the fields of emotion, motor control, electrophysiology and signal processing.

From a neurophysiological framework, depending on the pleasant or unpleasant emotional stimuli, Miller et al. (2019) highlighted distinct projections coming from the amygdala to sub-cortical hypothalamic pathways involved in approach and/or avoidance behaviors in rats. In humans, studies with functional magnetic resonance imaging, positron emission tomography or electroencephalography have revealed modulations in the motor inhibition by the amygdala depending on emotional contexts (Lane et al., 1997; Sagaspe et al., 2011; de Oliveira et al., 2012; Grèzes et al., 2014). Using transcranial magnetic stimulation, Coombes et al. (2009) reported that unpleasant context increases the cortical excitability of the primary motor cortex. More precisely, corticospinal excitability was modulated during the preparation of motor responses when viewing emotional pictures with different arousals, while movement speed and force production concomitantly varied as a function of the emotional valence of the pictures. Nevertheless, Blakemore and Vuilleumier (2017) wrote that “there has been a relative lack of effort to link affective neuroscience with movement neuroscience”. International Affective Picture System and reaching paradigms have been extensively used in their respective fields of emotion and motor control, but the novelty of combining the two in the present study thus represents a robust method for precisely defining the influence of viewing different categories of affective pictures on motor control.

To fill this gap, the present study investigates the neural mechanisms of movement control during the initiation of arm pointing movements according to the emotional context. To do so, the proposed protocol combined the viewing of standardized affective emotional pictures (IAPS) with the realization of a well-known motor pointing task (Bonnetblanc et al., 2004; Fautrelle et al., 2010a,c). Such hand pointing movements are performed from an upright standing posture and allow investigating the neural mechanisms of the CNS in the control of voluntary movements (Bernshtein, 1967; Goodale and Milner, 1992; Massion, 1992; Vernazza-Martin et al., 1999). Indeed, to point a target with their arm from standing position, the CNS has to manage two objectives: keep the balance of the body so that it does not fall, while moving the hand toward the spatial position to be pointed. To do so, the first muscles to be activated are not arm muscles but postural muscles in the leg and the trunk (Fautrelle et al., 2010c). These postural muscle contractions, which take place before the beginning of the arm movement, are called anticipatory postural adjustments (APA) (Massion, 1992). During this anticipation phase, the arm is not lifting but absolutely still. The APA and the beginning of the movement [from about 80 ms (Fautrelle et al., 2010a,c)] of the postural muscle activations shows that they cannot be triggered reflexively via afferent loops induced by the hand pointing movement, and do emanate necessarily from a programmed central command (Massion, 1992). The efficiency of this programmed central command and thus of the APA are well-known to be decisive for the movement performance, in stabilizing balance and posture, facilitating arm movements, and allowing coordination between posture and movement (Massion, 1992; Stapley et al., 1998; Kitaoka et al., 2004). The application prospects of this study could plead for the developments of innovative protocols in rehabilitation for patients with motor dysfunctions, in elite sport training and motor performance in extreme environments, or in motor learning protocols (Coudrat et al., 2014; Blakemore et al., 2018; Yoshida et al., 2019). This is why we started by studying and investigating to better understand the impact of emotional context on the programmed central command [from the APA (Massion, 1994) until the very first occurrences of the retroactive sensory-motor loops engaged by the realization of the movement (Fautrelle and Bonnetblanc, 2012)], although the study of the potential impact of emotional context on the feedback loops would be also an interesting next perspective. Many parameters can influence the motor command responsible for performing hand pointing movements. However, these parameters such as fatigue (Schmid et al., 2006), the difficulty index (Fautrelle et al., 2011) or even the participant's possibilities of anticipation are well known and are all controlled in the present study in order to have only one experimental variable manipulated: the affective context.

In this study, the motivational direction model - associating emotions with behavioral tendencies of approach or avoidance (Lang, 1995) - was used to investigate how emotional pictures modulate the behavioral parameters of the hand movement. Participants were required to point toward targets while viewing pleasant, neutral, or unpleasant emotional affective pictures from the IAPS. It is worth saying that the motor task pointing toward targets placed in front of the participants involves an approach movement. However, the experimental set-up was not designed to have pleasant or unpleasant outcomes, and pressing the button on the illuminated target did not cause any particular effect [for example: the disappearance of the emotional picture as used in Vernazza-Martin et al. (2017)] Therefore, and in line with the motivational direction model that we chose to use (Lang, 1995), the present behavioral hypothesis was that the times for the hand movement to reach the targets would be longer when viewing unpleasant pictures compared to when viewing pleasant ones.

From the perspective of motor control, the CNS has to manage at the kinematic, articular angle, musculoskeletal, and motoneuronal stages, with a high level of redundancy in the degrees of freedom, i.e., an infinite number of possibilities at each of the above stages to control the voluntary movement (Bernshtein, 1967; Jeannerod, 1986a,b). Consequently, there is an infinite amount of motor program content available for performing the movement, providing flexibility in motor control but making it particularly complex (Berret et al., 2009; Fautrelle et al., 2010b). Even though this issue is still a vast field of investigation, the CNS seems to solve such redundancy by grouping the regulation of muscle activities in more global functional units: this is the muscular synergy principle (Bernshtein, 1967; Ivanenko et al., 2005; Torres-Oviedo et al., 2006). For this reason, Farmer et al. (2007) suggested that synergistic muscle motoneurons share common corticospinal drives, resulting in intermuscular coupling as quantified by the oscillatory synchronicity of electromyographic (EMG) activity in synergistic muscle pairs - the so-called “intermuscular” (or EMG-EMG) coherence (IMC). IMC thus allows both considering the implication of a common central command on the moto-neuronal pools responsible for muscle recruitment (Kattla and Lowery, 2010), and investigating the patterns of coordinated neural drive responsible for the intermuscular coordination.

Thereby, IMC analysis provides a valuable methodological opportunity to better understand the impact of viewing emotional pictures on the contents of the motor command sent by the CNS to the muscles during the initiation of a voluntary pointing movement. In view that intermuscular coherence in the beta [β, (15–35) Hz] and gamma [γ, (35–60) Hz] bands can be thought to reflect the shared neural inputs from the motor cortex to the muscles during voluntary contractions (Kattla and Lowery, 2010), we adopted an exhaustive and exploratory approach by computing IMC to explore to which extent whole-body patterns of muscular activations are affected by emotional context. We hypothesized that the expected increase in hand movement times to point the targets when viewing unpleasant pictures could be explained, at least partly, by significant modulations of intermuscular coherence values. For example, from a neurophysiological point of view, two potential hypotheses could be made: in the first one, the IMC between the postural muscles and the focal muscles would decrease in unpleasant conditions compared to neutral and pleasant ones, generating an overall slowing down of the movement produced. In a second one, the IMC between the muscles would significantly increase in pleasant conditions allowing a shorter movement time compared to neutral and unpleasant ones. In other words, we hypothesized modulations of neural mechanisms of motor control during the initiation of arm pointing movements, as highlighted by significant differences in the intermuscular coherence, depending on the affective emotional picture conditions.

## 2 Material and methods

### 2.1 Participants

Sixteen right-handed participants took part in the experiment (8 men, 8 women, 24.4 ± 3.5 years old, height 175.7 ± 3.9 cm). Inclusion criteria were: (1) being between 18 and 29 years old; (2) being affiliated to the French social security system and having health insurance; (3) height between 170 and 185 cm; (4) being right-handed (Oldfield, 1971); (5) not having any musculoskeletal disorders or recent musculoskeletal injuries; (6) not having known phobia; (7) no neurological history; (8) not following acute or chronic drug treatment; (9) having a normal or corrected to normal vision; (10) not consuming neuro-stimulants or energy drinks 24 h before the experience. Phobia were investigated by open interview and laterality by the Edinburgh inventory (Oldfield, 1971). Exclusion criteria: (1) non-compliance with one of the inclusion criteria plus; (2) carrying out a professional or semi-professional activity of intervention in hostile situations or rescue involving the potential experience of violent visual scenes (firefighter, soldier, war-reporter, etc…); (3) having already viewed these images from the IAPS (in a previous scientific experiment for example). This method of sampling with men and women for such kind of study investigating the effects of emotional modulations on motor skills has already been published several times by our groups and other research groups (Gélat et al., 2011; Vernazza-Martin et al., 2015, 2017).

No explicit information was given about the purpose of the study before the experiment. All participants gave written informed consent to participate in the experiment. All procedures were approved by the Ethics Committee of the Federal University of Toulouse Midi-Pyrénées (N°: IRB00011835-2020-04-21-212) and complied with the Declaration of Helsinki as of 2008.

### 2.2 Experimental procedure

**Figure 1:**
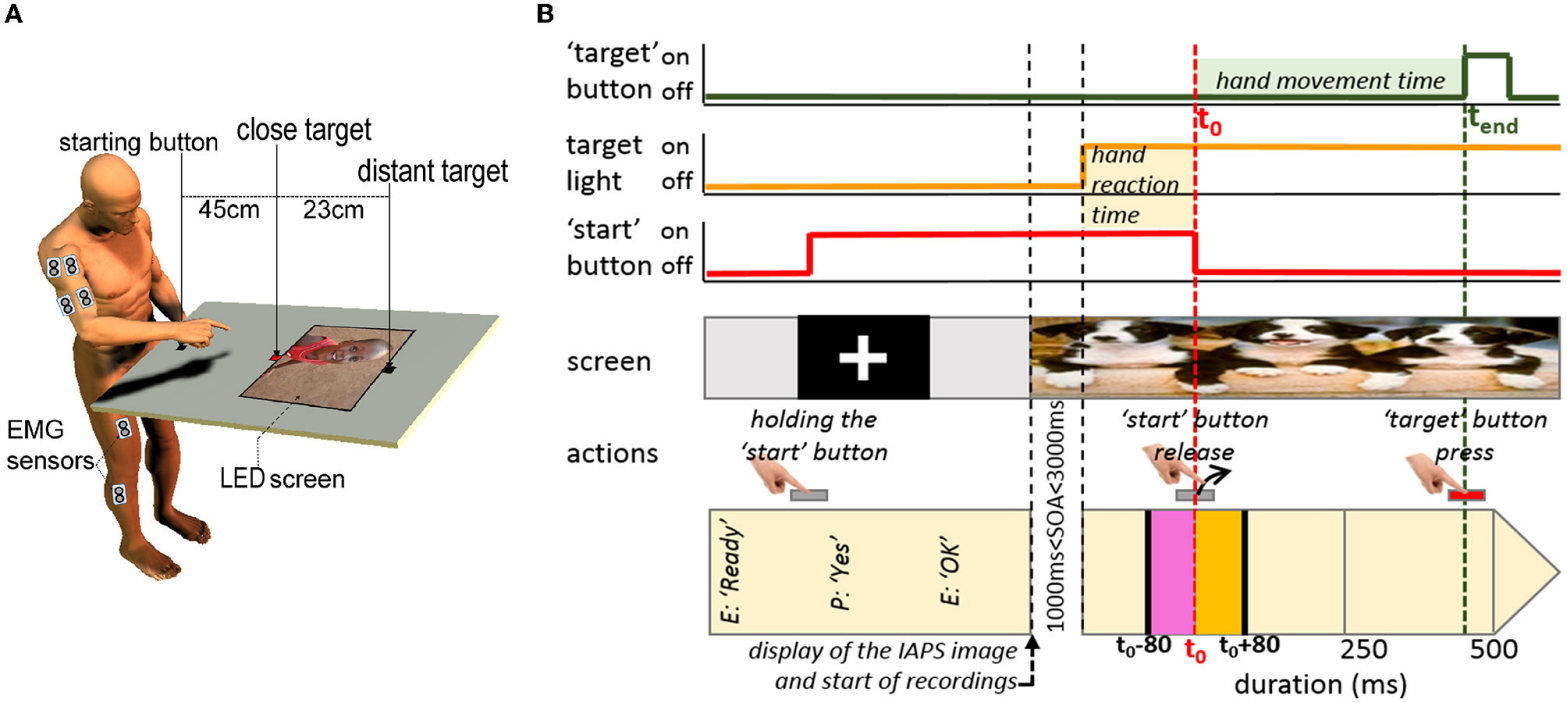
**(A)** Illustration of the experimental set-up for the pointing task. From the ‘start' button, participants were asked to point their hand at the target (close or distant) as quickly as possible as soon as it lit up. **(B)** Schematic representation of the recording process and hand movement parameters from typical raw data. The first three “curves,” respectively represent: in green the signal of the “target” button under the target light pressed at the end of hand movement (t_end_), in orange the signal of the “target” light ignition, and in red that of the “start” button held in the initial position and released at the hand movement onset (t_0_). Hand movement reaction time was the duration between “target” light ignition and t_0_; hand movement time was the duration between t_0_ and t_end_. The fourth row shows the display on the screen during the different phases of a typical pointing movement: a fixation cross was presented during the time period corresponding to the gray horizontal rectangle; an IAPS picture during the time duration of the green one. The fifth row shows the motor actions carried out by the participant. The last row is the time-lapse, with “E” and “P” being the abbreviations for “experimenter” and “participant,” respectively. Intermuscular coherence analysis was performed over both the [t_0_ - 80:t_0_] period corresponding to the APA (pink rectangle), and the [t_0_:t_0_ + 80] period corresponding to the beginning of the hand movement (orange rectangle).

Participants performed pointing movements with their right hand. The starting button (a tactile 10 × 10 mm square switch which allowed accurate measurement of the time-to-starting button release), the close and the distant targets were located 20, 65, and 88 cm respectively in front of the participant's sagittal plane and 15 cm below the xyphoid process. Consequently, the distance between the starting button and the close target was 45 cm, and the distance between the starting button and the distant target was 68 cm. The close target could be pointed with just an arm movement, whereas pointing the distant target required an additional forward trunk bending movement. Targets were represented by small visual and tactile 10 × 10 mm square switches which could be lit up (red color, 1 mcd in luminance) and which allowed accurate measurement of the time-to-target contact (please see the *Reaction Times and Movement Times* paragraph). A LED screen (380 × 213 mm, 60 Hz, 1,920 × 1,080 pxl) was inserted between and tangent to both targets and allowed displaying pictures in the visual field of the participants watching the targets (see Figure 1A). For each trial, a gray fixation cross on a black background was first displayed in the center of the screen. The instruction given to the participant was to “fix the central cross and keep pressed the starting button.” Then for each trial, when the participant said it was ready, a pleasant, neutral or unpleasant affective emotional picture coming from the International Affective Picture System (IAPS) database replaced the gray fixation cross (please see the *Emotional Pictures* paragraph). The instruction given to the participant was to fix the displayed picture and always keeping the starting position. After a Stimulus Onset Asynchrony (1-3s SOA), one of the two targets was lit up. Participants were asked to perform pointing movements “as quickly and as accurately as possible when a target was illuminated,” in six experimental conditions (2 Target Distances × 3 Affective Picture Content stimuli). A total of 90 movements per participant (15 movements × 6 experimental conditions) were randomized. Before data recording was initiated, each participant performed six-movement tests which always occurred in the following order: three tests in the “close × neutral” condition, three tests in the “distant × neutral” condition (here, the neutral picture was always the same - number 7705 in the IAPS - and was not used later during data acquisition).

### 2.3 Reaction times and movement times

In the initial position, the close and distant targets were represented by small, visual, and tactile 10 × 10 mm square switches, which could be lit up and allowed accurate detection of time-to-contact. They sent analog signals at a 1,000 Hz sampling frequency. Classically in the field of motor control literature for this kind of pointing task, the beginning of the movement is materialized by the beginning of the kinematic movement of the effector. In this study, the beginning of the hand movement, called hand movement onset (t_0_), is thus detected by the tactile switch. From there, the reaction time was computed as the duration between the moment at which a target was lit up and the hand movement onset (t_0_). The movement time was computed as the time lapse between the hand movement onset (t_0_) and offset (t_end_) (see Figure 1B).

### 2.4 Emotional pictures

The emotional pictures were chosen based on their affective valence and arousal. The objective was to select pictures with affective valence scores as high as possible for the pleasant condition, as neutral as possible in the neutral condition, and as low as possible for the unpleasant condition, and this for both male and female participants as previously published (Vernazza-Martin et al., 2017). Moreover, potential effects of arousal on posture and movements were carefully prevented by choosing pictures with the highest pleasant arousal, the highest unpleasant arousal, whereas neutral pictures were based on the recommendations specified by Bonnet et al. (1995) and Horslen and Carpenter (2011).

Consequently, the affective emotional stimuli used to induce emotional states during the voluntary arm pointing movements comprised 45 digital pictures for each gender selected from the IAPS database. The 15 pleasant pictures were selected from numbers 4,607, 4,611, 4,641, 4,647, 4,650, 4,651, 4,652, 4,658, 4,659, 4,680, 4,687, 4,690, 4,694, 4,695, 4,750, 4,800, 4,810 (description: erotic female, erotic couple, nude, and romance; valence: 8.00 ± 1.02; arousal 7.00 ± 0.3) for the men and from numbers 1,540, 1,710, 2,216, 4,626, 5,621, 5,910, 7,502, 8,030, 8,080, 8,185, 8,190, 8,200, 8,370, 8,470, 8,490, 8,496 (description: family, children, wedding and puppies; valence: 7.98 ± 0.45; arousal 6.74±0.55) for the women. The 15 neutral pictures were selected from numbers 1,080, 1,101, 1,303, 1,321, 1,390, 1,617, 1,932, 2,220, 2,704, 3,210, 3,302, 5,940, 6,900, 6,940, 7,004, 7,009, 9,411 (description: animals and dishes; valence 5.00 ± 0.24; arousal 5.2 ± 0.44) for the men and from numbers 1,303, 1,560, 2,780, 3550.2, 4,647, 4,649, 4,651, 4,664, 4,669, 4,683, 5,920, 5,950, 7,010, 7,640, 8,160, 8,192 (description: animals, sport scenes; valence 5.02 ± 0.44; arousal 4.95 ± 0.40) for the women. The 15 unpleasant pictures were selected from numbers 3,000, 3,010, 3,060, 3,069, 3,071, 3,080, 3,130, 3,170, 3,400, 3,500, 3,530, 6,230, 6,260, 6,350, 9,300, 9,405, 9,810 (description: mutilations, dead body, aimed gun); valence 1.9 ± 0.42; arousal 7.01 ± 0.16) for the men and from numbers 3,000, 3,010, 3,030, 3,053, 3,063, 3,064, 3,068, 3,069, 3,080, 3,100, 3,102, 3,120, 3,130, 3,170, 9,410 (description: mutilations, baby tumors, dead body; valence 1.16 ± 0.20; arousal 8.1 ± 0.45) for women. The scores of the computerized 9-point version of the Self-assessment Manikin (SAM) scale were used to control the 15 pleasant, neutral and unpleasant pictures in the affective dimension according to gender (Smith et al., 2005). An evaluation session was conducted for each participant at least 48 h before the experimental session to ensure that the affective perceptions of each picture in each volunteer participant were really in accordance with the expected experimental condition. To attest and validate the picture selections, univariate analyses were conducted in order to test whether the valence and arousal ratings significantly differed between the pleasant, neutral, and unpleasant picture subsets. These statistical analyses clearly showed a valence rating effect (*F*_(2, 87)_ = 2069.5, *p* < 0.001, η^2^ = 0.99). *Post-hoc* analyses revealed that valence ratings significantly differed between the three conditions pleasant/neutral, pleasant/unpleasant, neutral/unpleasant (all *p* < 0.001, all η^2^ = 0.96). An arousal rating effect was also revealed (*F*_(2, 87)_ = 69.9, *p* < 0.001, η^2^ = 0.74). Post-tests revealed that arousal ratings differed for the neutral/pleasant and neutral/unpleasant comparisons (both *p* < 0.001, η^2^ = 0.84) but not between the pleasant and unpleasant subsets (*p* = 0.52). Immediately after the experiment, another control evaluation using the 9-point version of the SAM, similar to the prior evaluation session, was performed to re-evaluate each picture in each participant. This re-evaluation aims to check that the double visualization has not significantly varied the affective perception of the images by the participants. [Valence; Arousal] scores of this control evaluation for the whole sample of participants were in average ± SD: [7.96 ± 0.61; 6.89 ± 0.48] in the pleasant subset; [5.10 ± 0.56; 4.95 ± 0.42] in the neutral subset; and [1.54 ± 0.68; 7.34 ± 0.28] in the unpleasant subset. No difference was observed in these intra-participant repeated measures comparisons between the emotional evaluations of the 45 selected pictures 48 h before the experimental session and the re-evaluation immediately after (all *p* > 0.64).

### 2.5 Surface electromyography

**Figure 2:**
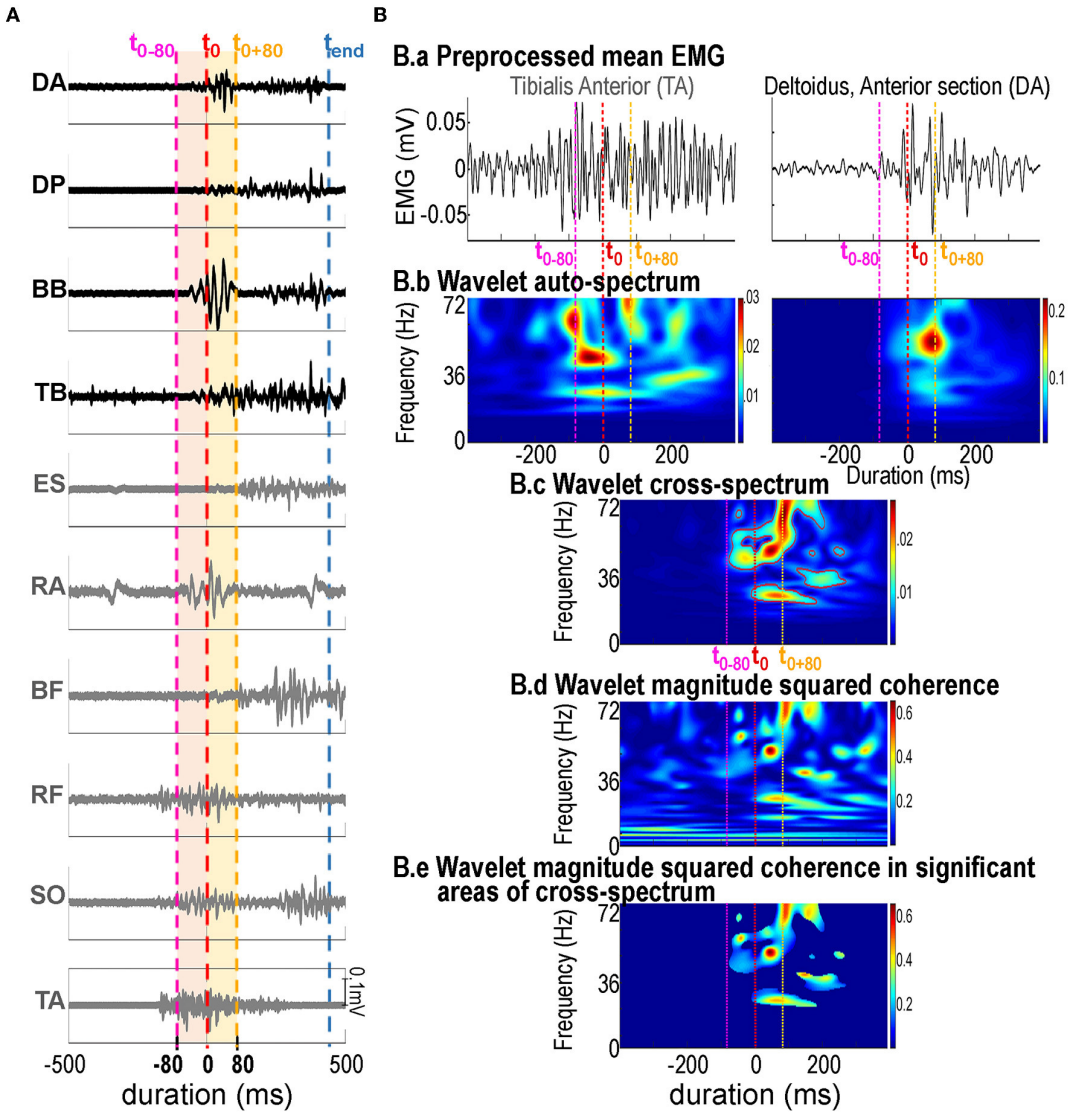
**(A, B)** Dashed vertical lines represent the occurrence of t_0_ (i.e., “start” button release, in red), t_0_-80 and t_0_ + 80 (80 ms before and after t_0_, respectively, in pink and orange) and of t_end_ (i.e., ‘target' button press, in blue). **(A)** Typical bandpass filtered (3–100 Hz zero-lag 4th order Butterworth filter) unrectified EMG signals of the ten recorded muscles from 500 ms prior to hand movement onset until 500 ms after. EMG signals from mainly postural muscles with a mainly focal role in the experimental task (DA, DP, BB, TB) are represented in black, those from mainly postural muscles (ES, RA, BR, RF, SO, TA) are represented in gray. Intermuscular coherence analysis was performed over both the [t_0_-80:t_0_] period corresponding to the APA (in pink), and the [t_0_:t_0_ + 80] period corresponding to hand movement initiation (in orange). **(B)** Illustration of the different processing steps used for time-frequency intermuscular coherence analysis. For each participant and in each experimental condition, intermuscular coherence (IMC) across 15 performed movements was calculated. ***B.a:*** mean EMG signals of a typical participant from TA (left column) and DA (right column). ***B.b:*** wavelet auto-spectra of EMG time-series from TA (left) and DA muscles (right). ***B.c:*** wavelet cross-spectrum between the two EMG time-series from TA and DA; the red contours identify the areas in the time-frequency plane where the correlation between the EMG signals is significant. ***B.d:*** wavelet magnitude-squared coherence between the two EMG time-series from TA and DA. ***B.e:*** wavelet magnitude-squared coherence between the two EMG time-series where the correlation between the EMG signals was detected significant on the wavelet cross-spectrum.

Surface EMG activities of 10 muscles were collected using an EMG system (Cometa, Milan, Italy) at a sampling frequency of 2,000 Hz. After appropriate skin preparation to reduce skin impedance below 5 kΩ (Hermens et al., 2000) surface electrodes were placed on the belly of each muscle parallel to the muscle fibers with an inter-electrode distance of 24 mm on the participant's right side for the tibialis anterior (TA), soleus (SO), rectus femoris (RF), and the biceps femoris (BF) in the leg area, the erector spinae (ES) between D11 and L1 and the anterior portion of the rectus abdominis (RA) at the trunk level, and the anterior section of the deltoïdus (DA), the posterior section of the deltoïdus (DP), biceps brachii (BB), triceps brachii (TB) on the arm (see Figure 2). Based on (i) the main aim of this study which was to investigate the neural mechanisms of movement control during the initiation of arm pointing movements according to the emotional context and (ii) our previous work showing that in such arm pointing movements, the first muscles to be activated by the CNS are the TA, followed by the DA and the BB muscles (Fautrelle et al., 2010a,b,c; Kubicki et al., 2016), the corresponding muscle pairs were classified into three groups:

- The “Posturo-Postural” group (PP) comprised the muscle pairs formed only with muscles considered as mainly postural in this particular motor task: TA-RF, TA-SO, TA-BF, TA-ES, and TA-RA.
- The “Foco-Focal” (FF) group comprised the muscle pairs formed only with muscles considered as mainly focal in the present motor task: DA-DP, DA-BB, DA-TB, BB-DP, and BB-TB.
- The “Posturo-Focal” (PF) group comprised the muscle pairs formed by a mainly postural muscle and a focal muscle TA-DA, TA-DP, TA-BB, TA-TB, DA-SO, DA-RF, DA-BF, DA-RA, DA-ES, BB-SO, BB-RF, BB-BF, BB-RA, and BB-ES.

### 2.6 IMC analysis

IMC was calculated as previously described in detail (Charissou et al., 2016, 2017) (see Figure 2B for illustration of the processing steps). Briefly, for each of the 24 selected muscle pairs, IMC was computed in the time-frequency domain using the *WavCrossSpec* (Bigot et al., 2011) from a band-pass filtered (3–100 Hz, zero-lag, 4th order Butterworth filter) unrectified EMG signal. Amid the debate on the relevance of EMG rectification in calculating corticomuscular or IMC, we made the decision not to rectify the EMG signals to avoid possible bias in IMC computation (McClelland et al., 2014). Parameters “nvoice” (wavelet scale resolution), “J1” (number of scales), and “wavenumber” (Morlet mother wavelet parameter) were, respectively, set at 7, 50 and 10 to accurately identify oscillatory activities in the [0.32·10^−2^: 0.23: 79.9] Hz frequency range.

In each frequency band of interest, i.e., beta, [β, (15–35) Hz] and gamma [γ, (35–60) Hz], IMC was quantified as the volume in the time-frequency plane under magnitude-squared coherence where the correlation between the EMG time series was significantly detected on the wavelet cross-spectrum (Bigot et al., 2011).

### 2.7 Time periods to perform the IMC analyses

IMC quantification in both frequency bands was performed on two-time windows of interest to specifically investigate the initiation of the voluntary movement.

- First: the [t_0_-80: t_0_] period. This time-period corresponds to the 80 ms before the hand movement onset. Indeed, it is well established that some postural muscle activities must precede voluntary pointing movements of the upper limb. These phenomena are called APA (Massion, 1992) and their anticipatory nature [from about 80 ms (Massion, 1992)] shows that they cannot be triggered reflexively via afferent loops induced by the hand pointing movement, and do emanate necessarily from a programmed central command (Massion, 1992).
- Second: the [t0:t0 + 80] period. This time-period corresponds to the very beginning of the movement initiation. Our previous studies in the field of motor control on this kind of pointing movement have demonstrated that the fastest possible feedback EMG modulation latencies were in the order of 100 ms (Desmurget and Grafton, 2000; Fautrelle et al., 2010c). By choosing an analysis time window of 80 ms, we make sure to analyze an EMG content without, or with very few online controls of the hand, but rather emanating necessarily from a programmed central command (Desmurget and Grafton, 2000; Fautrelle et al., 2010c) (see Figures 1B, 2).

### 2.8 Statistical analyses

The normality of the data distributions was tested by Shapiro-Wilk W-tests and the equality of sphericity by Mauchly's tests. When the data did not follow normal distributions even after log-transformation, Friedman chi-squared tests were conducted to investigate Target Distance effect and Affective Picture Content effect. When data followed normal distributions and showed equality of sphericity, ANOVA with repeated measurements were conducted. For the analyze of the movement times, a two-way ANOVA with repeated measurements were conducted to test the 2 Target Distance (close/distant) × 3 Affective Picture Content effects. Bonferroni *post-hoc* analyses were then conducted when necessary. For the analyze of the EMG data, a three-way ANOVA with repeated measurements were conducted to test the effects of (i) Target Distance (close/distant), (ii) Affective Picture Content (pleasant/neutral/unpleasant) and (iii) Time-Frequency Area [βIMC on (t_0_ – 80:t_0_); βIMC on (t_0_:t_0_ + 80); γIMC on (t_0_ – 80:t_0_); γIMC on (t_0_:t_0_ + 80)], separately for each group of muscle pairs. Bonferroni *post-hoc* were then applied when necessary.

## 3 Results

The hand movement reaction time and movement time (Figure 1B) and surface EMG activities of 10 muscles (Figure 2A) were recorded concomitantly during voluntary pointing movements. These movements were initiated from a standing posture, toward a close or a distant visual target (Figure 1A) while a picture was presented along the horizontal component of the point movement. Six conditions were tested: 2 Target Distance (close/distant) × 3 Affective Picture Content (pleasant/neutral/unpleasant). All numerical results are expressed as the mean ± standard deviation (SD); all graphical results are presented as the mean ± standard error (SE).

### 3.1 Hand movement time is longer in an unpleasant affective context than in a pleasant one

**Figure 3:**
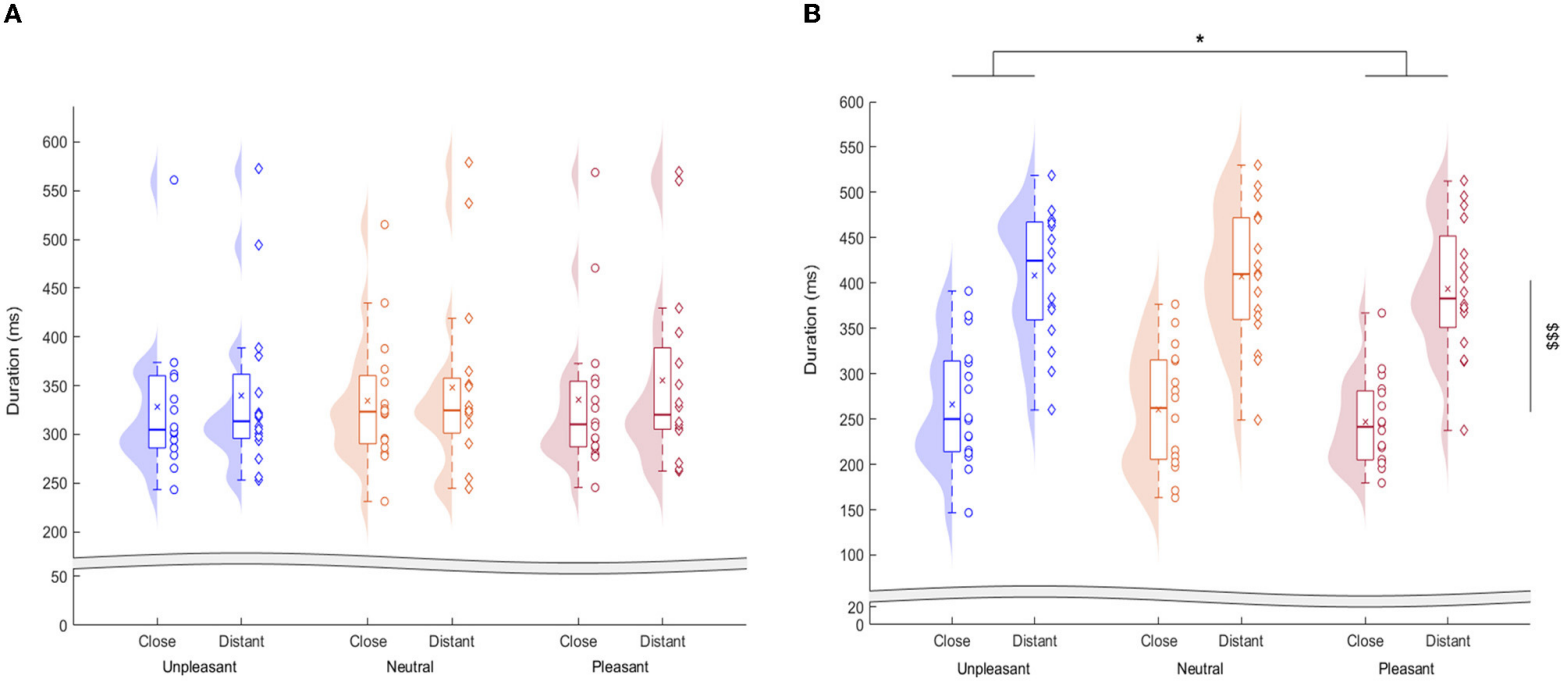
Reaction times **(A)** and hand movement time **(B)** in the three emotional picture contexts for both target distances. Central horizontal bars on boxplots indicate median values, crosses indicate mean values, boxes represent first through third quartiles, and lower and upper ends correspond respectively to the minimum and maximum values. *indicate the Affective Picture Content effect (*p* < 0.05) and $$$ indicate the Target Distance effect (*p* < 0.001).

Reaction time values (Figure 3A) did not follow a normal distribution even after log-transformation (Shapiro-Wilk W-tests; *P* < 0.01). Friedman chi-squared tests were conducted and did not reveal any significant Target Distance effect (χ^2^= 3.5, *p* = 0.36) or Affective Picture Content effect (χ^2^ = 2.3, *p* = 0.10).

Hand movement time values (Figure 3B) followed a normal distribution (Shapiro-Wilk tests, *p* > 0.46) and showed equality of sphericity (Mauchly's tests, *p* > 0.37). Two-way ANOVA with repeated measurements were conducted and revealed a significant Target Distance effect (*F*_(1, 15)_ = 167.70, *p* < 0.001; η^2^ = 0.91), a significant Affective Picture Content effect (*F*_(2, 30)_ = 4.12, *p* < 0.05; η^2^= 0.30), but no interaction effect (*F*_(2, 30)_ = 0.14, *p* = 0.87). Bonferroni *post-hoc* analysis revealed that hand movement time was significantly longer in an unpleasant context than in a pleasant one (0.35 ± 0.10 s *vs*. 0.33 ± 0.10 s, *p* < 0.05). Hand movement time in the neutral context (0.34 ± 0.11 s) was not significantly different from that in a pleasant or an unpleasant context (*p* = 0.62 and *p* = 0.63, respectively).

### 3.2 IMC in postural muscle pairs is lower in an unpleasant context than in neutral and pleasant ones

βIMC and γIMC values followed a normal distribution (Shapiro-Wilk tests, *p* > 0.33) and showed equality of sphericity (Mauchly's tests, *p* > 0.21). Three-way ANOVA with repeated measurements were conducted to test the effects of (i) Target Distance (close/distant), (ii) Affective Picture Content (pleasant/neutral/unpleasant) and (iii) Time-Frequency Area [βIMC on (t_0_ – 80:t_0_); βIMC on (t_0_:t_0_ + 80); γIMC on (t_0_ – 80:t_0_); γIMC on (t_0_:t_0_ + 80)], separately for each group of muscle pairs. Bonferroni *post-hoc* were applied when necessary.

**Figure 4:**
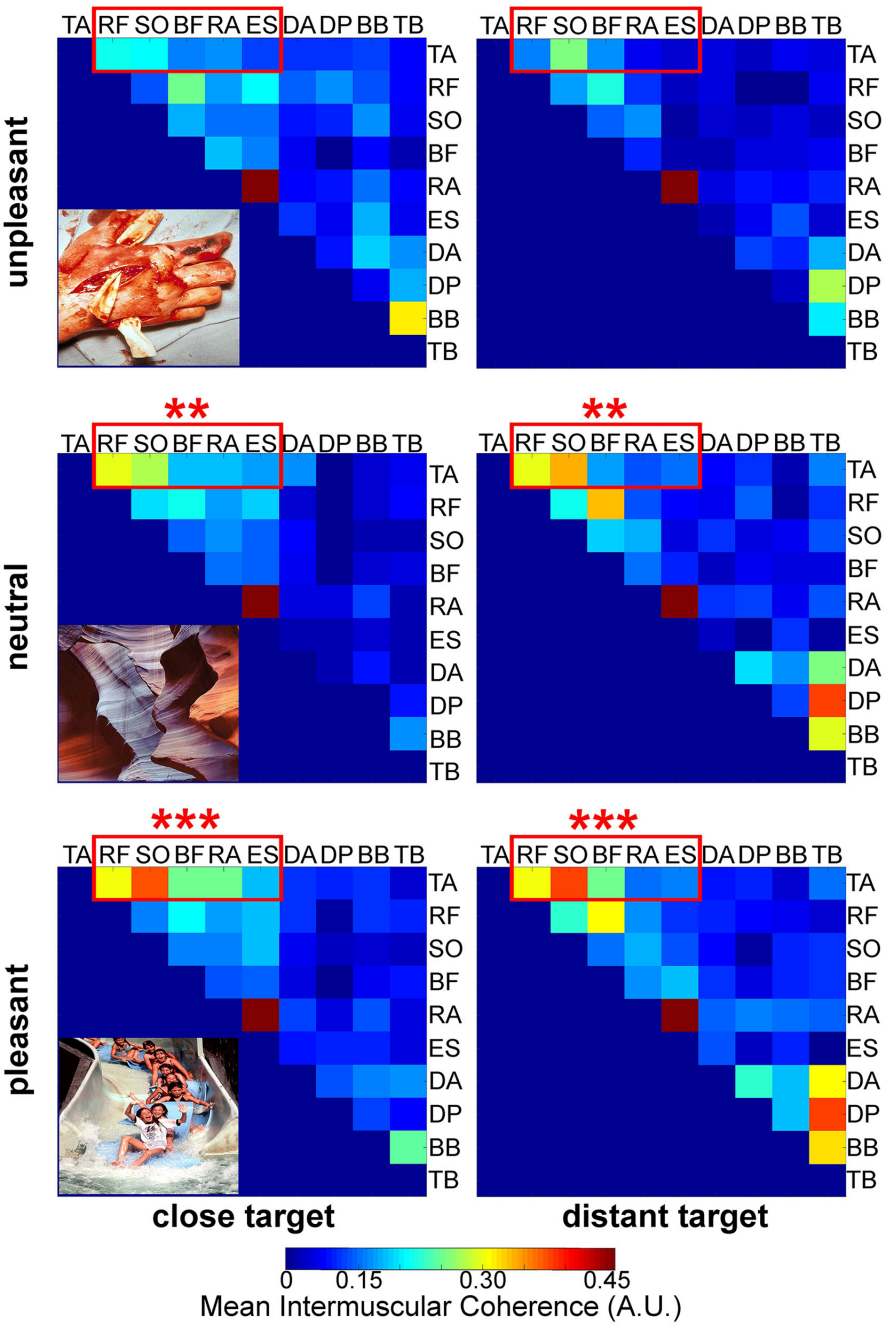
Pictorial representation of mean intermuscular coherence matrix (see upper diagonals) in all the muscle pairs studied in the three affective picture contexts (i.e., unpleasant, neutral and pleasant from the **top row** to the **bottom row**) and the two target distances (i.e., close and distant on the **left** and **right column**, respectively). TA, Tibialis Anterior; RF, Rectus Femoris; SO, Soleus; BF, Biceps Femoris; RA, Rectus Abdominis; ES, Erector Spinae; DA, Deltoidus, Anterior section; DP, Deltoidus, Posterior section; BB, Biceps Brachii; TB, Triceps Brachii. Muscle pairs of the “Posturo-Postural” group are framed in red. Statistical analyses were carried out for these posturo-postural muscle pairs: ** and *** indicate *p* < 0.01 and *p* < 0.001, respectively.

In the PP group of muscle pairs (please see paragraph Surface electromyography and Figure 4), the results showed a significant effect of Affective Picture Content (*F*_(2, 30)_ = 4.21, *p* < 0.05, η^2^ = 0.40), but no significant Target distance effect (*F*_(1, 15)_ = 0.12, *p* = 0.54, no Time-Frequency Area effect (*F*_(3, 45)_ = 2.49, *p* = 0.18) or interaction (*F*_(6, 90)_ = 0.70, *p* = 0.90). Bonferroni *post-hoc* analysis revealed that the mean IMC in muscle pairs of the PP group significantly decreased in the unpleasant context (0.12 ± 0.06 a.u.), compared to both neutral (0.15 ± 0.06 a.u.; *t*_(127)_ = 3.29, *p* < 0.01, η^2^= 0.23) and pleasant (0.148 ± 0.062 a.u.; *t*_(127)_ = 4.06, *p* < 0.001, η^2^ = 0.33) contexts. No significant difference was found on IMC between the neutral and pleasant conditions (*t*_(127)_ = 1.16, *p* = 0.12*)*. No significant effect was highlighted on IMC in both PF (in average: *F* = 0.74, *p* = 0.84) and FF (in average: *F* = 0.11, *p* = 0.54) muscle pair groups.

### 3.3 Movement time and IMC in posturo-focal muscle pairs are negatively associated in an unpleasant context

**Figure 5:**
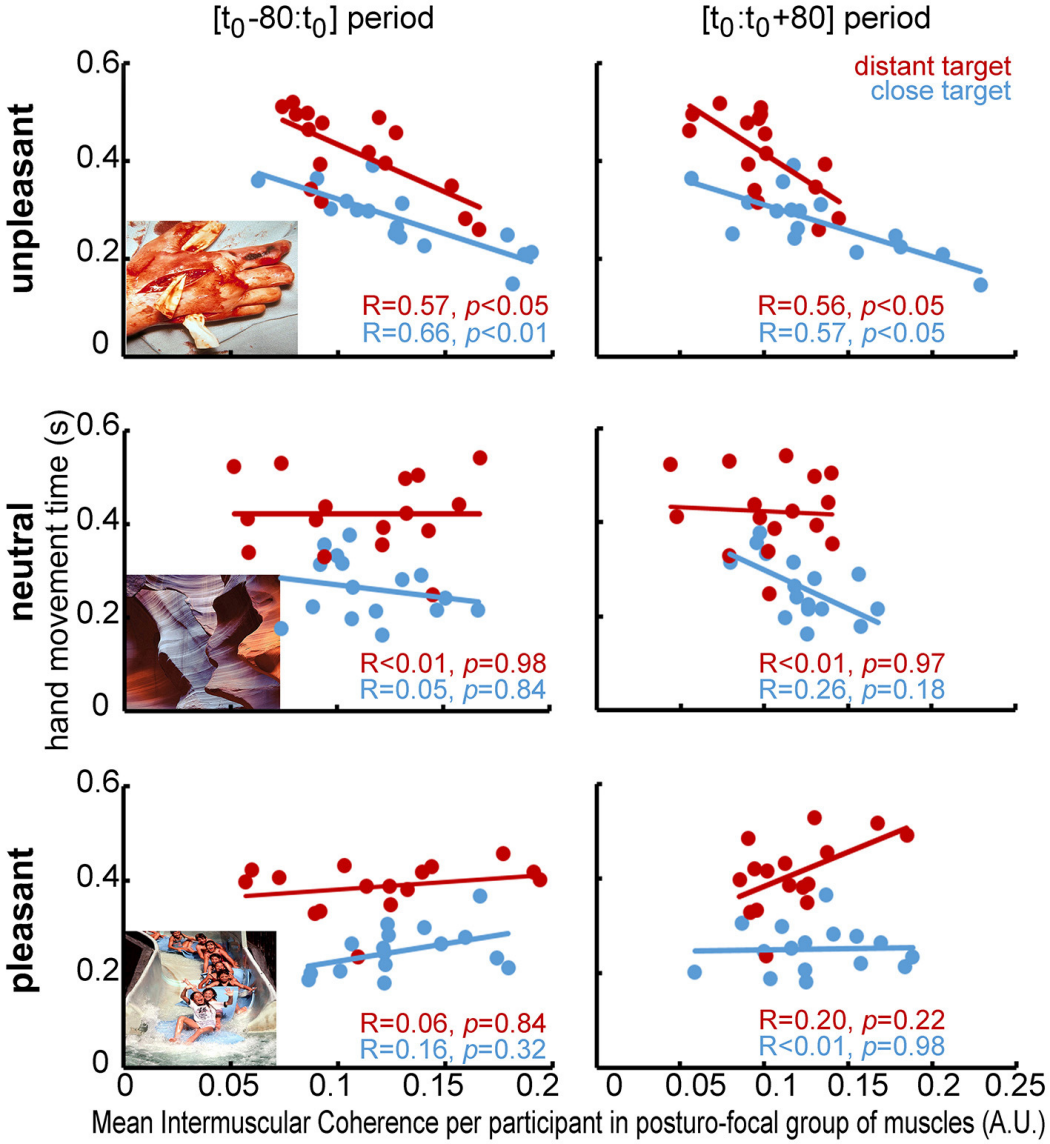
Linear regressions between movement time and βIMC in the two target distance conditions (close target condition in blue, distant target condition in red) in the “Posturo-Focal” group of muscle pairs during the [t_0_ - 80:t_0_] period (left column) and the [t_0_:t_0_ + 80] period (right column) in the three emotional picture contexts (i.e., unpleasant, neutral and pleasant from the **top row** to the **bottom row**). For each graph: the *x-axis* shows the βIMCs in arbitrary units (A.U.) of the posturo-focal muscle pairs for each participant in the two target distances.

Finally, the correlation relationship between hand movement parameters and IMC values in the groups of muscle pairs was studied for each target distance in each of the three emotional contexts involved (see Figure 5 for βIMC). These relationships highlighted significant negative correlations between the hand movement time and IMC in the PF group in each target distance in the unpleasant context only: significant linear correlations were found between hand movement time and βIMC during the [t_0_ - 80:t_0_] period (DoF = 14; *R* = 0.57, *p* < 0.05 and DoF = 14; *R* = 0.66, *p* < 0.01 in the close and distant target condition, respectively), and between hand movement time and both βIMC (DoF = 14; *R* = 0.56, *p* < 0.05 and DoF = 14; *R* = 0.57, *p* < 0.05 in the close and distant target condition, respectively) and γIMC (DoF = 14; *R* = 0.50, *p* < 0.05 and DoF = 14; *R* = 0.52, *p* < 0.05 in the close and distant target, respectively) during the [t_0_:t_0_ + 80] period. No other significant effect was highlighted in the neutral [DoF = 14; (max *R* = 0.13: min *R* < 0.001), *p* = 0.98 in average] or pleasant [DoF = 14; (max R = 0.11: min R < 0.001), *p* = 0.88 in average] context.

## 4 Discussion

In this study, we aimed to bring new insights into whether and how emotional pictures could modulate motor control mechanisms during the initiation of arm pointing movements. We carried out a multimodal analysis combining the use of a well-known visuomotor pointing task performed from a standing posture with standardized affective emotional pictures. The analysis focused on the assessment of reaction time, hand movement time, and IMC from EMG activities of 10 muscles located in various key places on the participants' bodies. Given that attentional levels and task difficulty are controlled and similar in every pointing movement condition, it is reasonable to consider that the measured effects in the present study can be solely attributed to affective context modulations.

### 4.1 Movement time is longer in an unpleasant context than in a pleasant one

On one hand, our results showed that hand movement time was longer when participants performed pointing movements toward visual targets in an unpleasant context compared to performing the same movements in a pleasant one. This behavioral result is partly in line with the motivational direction model and related findings from the psychology literature (Lang, 1995; Chen and Bargh, 1999). Indeed, according to the motivational direction model, approaching the hand toward a highly arousing pleasant picture produce a congruent situation that results in speeding up the movement compared to approaching a highly arousing unpleasant picture (Lane et al., 1997; Elliot and Thrash, 2002; Elliot et al., 2006; Lang and Bradley, 2010; Phaf et al., 2014). Noteworthy is that the effect of the unpleasant emotional context on motor behavior was small to medium and thus also in line with the existing literature. In short, the present results validate our initial hypothesis dealing with the modulation of the hand movement time by the emotional context.

On the other hand, our results do not show any significant difference in the reaction times based on the emotional context, which does not support our initial hypothesis. This being the case, the literature does not present a perfect consensus on approach-avoidance tendencies in manual reaction time tasks. Although some studies generally show a significant decrease in reaction time for approach movements toward pleasant stimuli, a publication bias emerged in a meta-analysis of these results (Phaf et al., 2014). Moreover, other studies did not show any significant decrease in reaction time when only the valence of the stimuli varied (Markman and Brendl, 2005; Rotteveel et al., 2015). In light of Phaf et al. (2014), three reasons may explain why no significant result emerged on the reaction times:

- First, the above-mentioned visuomotor task is pointing movement without any other goal than respecting the given instructions (a so-called intransitive movement). On the contrary, the other studies, which showed significant modulation of reaction times depending on the emotional context, used transitive motor tasks, i.e., pointing movements have here a functional goal such as turning off the projected pictures for example. Consequently, such studies dealt with the motivational direction theoretical model as well as with the affective meaning of the intended response goal model, which differs from the paradigm used in the present study.
- Secondly, the present study also differs from previous works using IAPS pictures in that the participants were given instructions about the movement velocity (i.e., “please perform every pointing movement as quickly and as accurately as possible” and “go point at the target as fast as possible as soon as the light appears”). It could have been hypothesized that the temporal pressure contained in the instructions given for the present study has had more impact on the reaction times than the emotional conditioning obtained by IAPS pictures visualization. At the opposite, using similar instructions about movement velocity but different facial expressions for emotional conditioning [happy and fearful faces in Mirabella (2018) vs. angry faces in Mancini et al. (2020)], recent studies demonstrated that reaction time is increased in unpleasant contexts as compared to pleasant ones. Therefore, exposing individuals to visual stimuli associated with different emotional content can significantly affect the kinematics of their subsequent movements.
- Finally, we used pictures coming from the IAPS database and displayed them on a 380^*^213 mm LED screen that was positioned about 75 cm from the participants' eyes. As the degree of commitment of the motivational system is related to the level of activation of the stimulus (Bradley et al., 2001a), we can hypothesize that the level of the present commitment may still not have been sufficient to impact the reaction times. Such a hypothesis seems corroborated by the recent findings of Calbi et al. (2022) and Mancini et al. (2022) which showed that also inhibitory control, a key executive function, is impacted by facial or body postures emotions respectively, but only when they are task-relevant. Finally, Mirabella et al. (2022) showed that whole-body movements share the same features as reaching arm movements regarding emotional stimuli, i.e., facial emotions altered reaction times and other kinematic parameters of the movements only when their conscious appraisal was requested.

Altogether, our results on hand movement parameters confirm previous findings for a whole-body movement (Vernazza-Martin et al., 2015, 2017), and are consistent with the motivational direction model. Nonetheless, this effect was moderate in size and significantly affected only the movement time. This need to be relativized somewhat considering the lack of effect of the IAPS on the hand reaction times and the moderate power effect of the IAPS on the hand movement times, which has nonetheless already been reported in previous studies (Yiou et al., 2014). When being faced with a highly arousing context, pointing toward a target in a pleasant emotional context creates a congruent situation, which would activate the appetitive neural circuits (Cacioppo et al., 1993; Lang, 1995; Chen and Bargh, 1999; Lang and Bradley, 2010) and speed up the movement compared to the case with an unpleasant context. On the contrary, pointing toward a target in an unpleasant emotional context creates an incongruent situation that would appear to slow down the movement compared to the situation in a pleasant context. Even if these behavioral results may be tempered by the fact that they do not show any significant difference between the neutral and unpleasant conditions, they are in line with our previous study (Vernazza-Martin et al., 2015, 2017) and somehow legitimize the proposed neurophysiological analyzes in order to better investigate the underlying mechanisms of the significant effect of emotional context on the EMG content of the muscular motor command. At any rate, these results support our second hypothesis that different emotional pictures could modulate the neural control mechanisms during the initiation of arm pointing movements.

### 4.2 IMC in postural muscle pairs decreases in an unpleasant context

To further explore these underlying mechanisms, we investigated whether and how pleasant, neutral or unpleasant pictures modulate the coupling between muscle pairs during the initiation of hand pointing movements. Concomitantly with the above change in hand movement time, our results on IMC showed that the coupling significantly decreased in the postural muscle pairs when the affective pictures presented were unpleasant.

Even if the absence of any Time-Frequency Area effect on IMC may be at least partly explained by the temporal smoothing of time-frequency analysis and further justifies to discuss our results without distinction between the [t0 - 80:t0] period and the [t0:t0 + 80] period, this original finding agrees with previous studies that highlighted the presence of synchronization between synergistic muscles during voluntary contractions, especially in the ß band (Boonstra et al., 2009; Poston et al., 2010), and supports our original hypothesis that intermuscular coupling is modulated by the emotional context.

This result may appear contradictory to those suggesting that intermuscular coherence is not associated with beta band coherence during dynamic tasks including dynamic reaching-like motion (Laine et al., 2021). However, beyond the methodological considerations that may explain such discrepancies between the results, our finding appears to be consistent with the literature showing synergistic temporal links in EMG activation delays during a visuomotor pointing task similar to that in the present study (Morasso and Schieppati, 1999; Fautrelle et al., 2010a), and that highlighted the ability of the CNS to modulate coupling links between muscles during arm movements. Indeed, the CNS can increase the muscular coupling during a corrective process with temporal emergency to alter the movement trajectory (Soechting and Lacquaniti, 1983; Fautrelle et al., 2010a) or answer some environmental or physical constraints (Berret et al., 2009), and decrease the postural muscle coupling when the CNS is degenerating, which is well known in frail elderly people or MCI patients (Kubicki et al., 2012, 2016).

Some authors emphasized that corticomuscular coherence may reflect the efferent drives involved in tonic contractions (such as postural contractions) and in high cognitive processes (such as attention focused on understanding emotional pictures) (Mima and Hallett, 1999; Mima et al., 2001). In our case, we could interpret the decrease in IMC as a decrease in synchrony of postural muscular activations because of the unpleasant emotional pictures. Indeed, the absence of significant modulation of IMC in muscle pairs with solely focal muscles could be interpreted as the CNS' strategy to modulate the functional coupling between posture and movement according to the emotional context. This is in line with previous findings reporting that the modulation of muscular coupling is very muscle-pair specific (Winges et al., 2006).

Moreover, some neurophysiological studies documented that being exposed to an unpleasant emotional context can induce negative shifts in the timing of two motor event-related potentials in the frontal cortex (Grecucci et al., 2009). Although this is somewhat speculative as we are dealing with EMG, it is possible that these negative shifts reported by Grecucci et al. (2009) may at least partly involved in the decrease of the synchrony in the EMG activities of the postural muscles in the unpleasant IAPS context. So, this result argues in favor of a possible cortical implication in the changes in patterns of muscular activations found in the present experiment.

### 4.3 Hand movement time is linked to IMC in posturo-focal muscle pairs

Our results highlighted a negative linear relationship between the magnitude of the IMC in posturo-focal muscle pairs and the hand movement time, but only when the emotional stimuli were unpleasant. In this context, the greater the magnitude of IMC, the shorter the movement time. This finding confirms that the ability of the CNS to synchronize postural and focal muscles in different ways could to be a factor in the hand movement times in an unpleasant emotional context, as previously suggested by earlier studies for whole-body reaching movements in anxious equilibrium conditions (Berret et al., 2009; Fautrelle et al., 2010b). Such modulation of intermuscular synchronization could also align with previous results on motor correction, showing that the intermuscular temporal coupling and the amplitude activation coupling in several pairs of posturo-focal muscles are significantly increased when the temporal pressure is high (Fautrelle et al., 2010a).

### 4.4 Limits

Notwithstanding the relatively small sample size of our study which could limit the generalization of the results, our findings are the first to provide strong preliminary evidence that longer movement times toward an unpleasant picture is associated to the decrease of the IMC coupling in the postural muscle pairs. The medium effects size we obtained with this relatively small sample reinforces the confidence on the behavioral and neurophysiological differences observed between the different affective contexts.

In this study, solely approach movements were investigated while avoidance movements were not. Consequently, the effect of IAPS in task-relevant and irrelevant conditions on the same participants was not carried out, that remains a limit for more general interpretations of these present results.

The use of the IAPS might reveal several limitations that must be considered when pursuing future studies. Firstly, the IAPS might be problematic because of the high heterogeneity of the pictures inside a same category of emotional valence. More precisely, showing a picture of a shark is very different from showing a human dead body picture, although they have both a negative valence. The differences between these visual features more than picture's valence might potentially influence the patterns of neural activation, including muscle specificity. Secondly, regarding the experimental design, the use of different categories of pictures for men and women could appear as a limit, since distinct emotional responding as a function of gender and categories has been described (Bradley et al., 2001b)^.^ In our study, our aim was to investigate the impact of the highest possible affective perceptions (including men and women) on the contents of the motor command sent by the CNS to the muscles during the initiation of a voluntary pointing movement. To do so, we had to use different categories of pictures for men and women in order to generate emotional stimuli among the most impactful possible for all participants. If we hadn't, we wouldn't have had matching in terms of arousal between men and women, and we would not have been in a normalized situation to analyze the contents of our muscular commands. Nevertheless, and in accordance with the first point above, the differences between these images for men and women could potentially influence the patterns of neural activation, including muscle specificity. This can potentially mix the effects of affective contexts on motor behavior and intermuscular coherence.

Future works will be allowed to study and then compare the specificity of the motor control of each gender with of identical stimuli but of different perceived arousal. Taken as a whole, the literature and the present results plead in favor of future studies investigating the impact of affective context on the control of human movement using set ups that are able to intensify the degree of motivational commitment of the participants. Additionally, hypothetical gender differences in the effects of emotions on motor control could be investigated.

### 4.5 Neurophysiological links and perspectives

Taken together, these results could be in line with the complex representation of muscular synergies in the primary motor cortex (M1). In “real life,” which is obviously difficult to reproduce in laboratory conditions, the ability to flee quickly when looking at snakes or guns pointed at your head are not just arbitrary stimuli but are totally relevant to survival (Ohman and Mineka, 2001; Rotteveel and Phaf, 2004). However, recent studies could let us hypothesize that emotional modulations exerted on M1 would rather desynchronize EMG activity in the muscles when performing an approach movement toward an unpleasant stimulus. Using transcranial magnetic stimulation and motor evoked potentials, some studies (Coombes et al., 2009; Nogueira-Campos et al., 2014) reported a higher neural excitability in M1 in an unpleasant emotional context, whereas, Hajcak et al. (2007) reported higher excitability for high arousal in pleasant and in unpleasant contexts. In the present study, the incongruent situation provoked by a fast pointing movement toward an unpleasant picture could activate the avoidance neural circuits (Duckworth et al., 2002) despite the need to perform an approach movement, inducing a decrease in the coupling of postural muscle activations. In line with the above studies, our results could be consistent with higher neural excitability in M1 inducing a stronger coupling in muscle pairs responsible for avoidance tendencies and, also, decreasing the coupling in muscle pairs responsible for approach tendencies.

A first perspective for deepening and strengthening the interpretation of the present results would be to investigate the influence of the emotional context on the IMC of avoidance movements. Moreover, several studies highlighted distinct involvement of neural pathways spanning from the amygdala to the hypothalamus (Miller et al., 2019), as well as different level of activation of the midcingulate cortex (Pereira et al., 2010; Lima Portugal et al., 2020) depending on the emotional context. The structural and functional connectivity between the amygdala and the motor areas appears crucial for affective processing (Pereira et al., 2010; Grèzes et al., 2014; Schönfeld and Wojtecki, 2019; Lima Portugal et al., 2020), and motor preparation for contextually relevant actions (Gilat et al., 2018; Pick et al., 2019). However, the impact of these changes in brain activations on the content of the EMG signal remains poorly understood in terms of oscillatory coupling (Schönfeld and Wojtecki, 2019). Indeed, considering that individual alpha motor neurons contributing to the EMG signal receive thousands of inputs from various sources including subcortical regions and spinal interneurons, the signal observed at the level of motor units cannot be used to infer activity from the motor cortex (Schieber, 2011). As a consequence, future studies using combined measures of brain and muscle activity (e.g., EEG and EMG) would be useful for deepening the understanding of the present results.

## Data availability statement

The raw data supporting the conclusions of this article will be made available by the authors, without undue reservation.

## Ethics statement

The studies involving humans were approved by Ethics Committee of the Federal University of Toulouse Midi-Pyrénées. The studies were conducted in accordance with the local legislation and institutional requirements. The participants provided their written informed consent to participate in this study.

## Author contributions

EP: Data curation, Investigation, Writing—original draft. CC: Data curation, Investigation, Software, Supervision, Writing—original draft. SV-M: Conceptualization, Supervision, Writing—review & editing. BP: Supervision, Validation, Writing—review & editing. RL: Supervision, Validation, Writing—review & editing. DA: Data curation, Investigation, Methodology, Software, Supervision, Validation, Writing—original draft, Writing—review & editing. LF: Conceptualization, Data curation, Funding acquisition, Investigation, Methodology, Resources, Supervision, Validation, Writing—original draft, Writing—review & editing.
